# Oral Candidal Colonization in Patients with Different Prosthetic Appliances

**DOI:** 10.3390/jof7080662

**Published:** 2021-08-16

**Authors:** Maja Kinkela Devcic, Suncana Simonic-Kocijan, Jelena Prpic, Igor Paskovic, Tomislav Cabov, Zoran Kovac, Irena Glazar

**Affiliations:** 1 Department of Oral Surgery, Faculty of Dental Medicine, University of Rijeka, Kresimirova 40/42, 51000 Rijeka, Croatia; maja.kinkela.devcic@fdmri.uniri.hr (M.K.D.); tomislav.cabov@fdmri.uniri.hr (T.C.); 2 Department of Prosthodontics, Faculty of Dental Medicine, University of Rijeka, Kresimirova 40/42, 51000 Rijeka, Croatia; zoran.kovac@icloud.com; 3 Department of Oral Medicine and Periodontology, Faculty of Dental Medicine, University of Rijeka, Kresimirova 40/42, 51000 Rijeka, Croatia; jelena.horvat.prpic@gmail.com; 4 Department of Agriculture and Nutrition, Institute of Agriculture and Tourism, K. Huguesa 8, 52440 Porec, Croatia; paskovic@iptpo.hr

**Keywords:** *Candida* species, oral colonization, oral lesions, prosthetic appliances, salivary flow rate

## Abstract

Oral infections caused by *Candida species* are becoming more common, which may be related to an increase in the number of immunologically compromised patients as well as favorable conditions in the oral cavity that often include removable prosthetic appliances. The purpose of this study was to determine the presence of a particular *Candida species* in patients with PMMA and Cr-Co prosthetic appliances, as well as the salivary flow rate, and oral signs and symptoms. This investigation included a total of 120 subjects with different kinds of removable dentures. A sample of concentrated oral rinse was collected from all subjects in order to detect *Candida* colonization and identify the *Candida* species, a quantum of salivation was measured, and subjects were examined clinically. *Candida* spp. was predominant among the subjects who were denture wearers (*p* < 0.0001). In all subjects, the most frequently detected species was *C.albicans*. A statistically significant difference was found between the prevalence of *C.albicans* (*p* < 0.001) and *C.krusei* (*p* < 0.001) in denture wearers. Subjects with PMMA-based removable prosthetic appliances mostly demonstrated a significant decrease in salivation (*p* < 0.001), an increase in burning sensations (*p* < 0.001), and dry mouth (*p* < 0.001) compared to the subjects who wore partial dentures with Co–Cr metallic frameworks. Red oral lesions were more frequently found among the subjects with partial dentures with Co–Cr metallic frameworks (*p* < 0.001). Regardless of the material used for the denture, patients must be regularly checked by their dentists in order to prevent the development of oral lesions.

## 1. Introduction

*Candida species* (*Candida* spp.) are one of the normal constituents of the physiological oral microbiota and may be found in 30–80% of healthy individuals [1,2,3]. Among them, *Candida albicans* appears to be the most common, accompanied by *Candida tropicalis*, *Candida glabrata*, *Candida parapsilosis*, *Candida krusei*, and *Candida pseudotropicalis* [1,4]. Although the positive finding of *Candida* in the oral cavity does not represent a pathological finding per se, this fungus has all the characteristics of the oportunistic pathogen, meaning it may cause an infection in the mouth when conditions arise. *Candida* may cause various forms of infections, from superficial to systemic conditions, which in extreme cases may lead to death [5]. The occurence of infection is influenced by factors altering the local oral environment as well as mucosal resistance. These include changes in the host’s immune system, administration of antibiotics, diabetes, smoking, advanced age, pregnancy, hyposalivation, inadequate oral hygiene, and various types of dentures [6,7,8,9]. Oral candidiasis poses a particular problem among cancer patients and patients on chemotherapy and radiotherapy. These are the main predisposing factors that compromise the immune system and put the patient at risk for oropharyngeal candidiasis [10]. Colonization with *Candida* spp. and the respective infections are also more frequent among transplant patients compared to non-transplant patients [11]. Furthermore, Candida is also closely associated with potentially malignant and malignant oral lesions [12]. According to the available evidence, candida cell counts greater than 600 CFU/mL in concentrated oral rinse samples are considered as a threshold for oral infection caused by *Candida* spp. [13,14]. Saliva is extremely important for the prevention of oral candidiasis. Lubrication, cleansing, hydration, and antimicrobial activity based on factors such as histidine-rich polypeptides, lactoferrin, lysozyme, and sialoperoxidase hinder the reproduction of *Candida* [15,16]. Therefore, the conditions leading to a change in quantity and quality of saliva create conditions for its reproduction and development of infection. This may cause development of lesions in the salivary glands and additionally reduce the salivary flow rate [15]. Moreover, it has been found that patients who are denture wearers are more predisposed to having their mucosa colonized by *Candida* spp. compared to the patients who are denture-free [5,17]. Removable dentures replace—beside the missing teeth—a fraction of the resorbed alveolar ridge and have a large contact area with oral mucosa, therefore creating the perfect conditions for unhindered reproduction of *Candida* in the narrow space between the denture and mucosa [3]. Materials used for manufacturing of the denture base are polymethyl methacrylate (PMMA) and cobalt–chromium alloys (Co–Cr). The type of materials used for prosthetic appliances can directly affect the presence of *Candida* in an oral cavity due to their properties (primarily the differences in surface structure, e.g., the degree of porosity, roughness, and surface-free energy which affects the adhesion of microorganisms and plaque formation). Appliances with a cast metal base manufactured from Co–Cr alloys have a more stable and smooth surface, and are not prone to development of surface porosity, such as PMMA-based materials, except in cases of non-intimate adhesion of the compound Co–Cr and PMMA on the basis of dentures which may act as a predilection site for fungal colonization [18]. Therefore, in cases where less stable materials are used, there is generally a greater risk of *Candida* growth and spread causing the clinically manifested oral infection. Knowledge of the factors contributing to disease development facilitates the prevention and reduces the occurence of infection [5]. Available evidence shows the correlation between the presence [quantity] of *Candida* spp. and the occurence of clinical oral signs and symptoms [19]. Symptoms reported by patients commonly include dry mouth, altered taste, and red lesions of oral mucosa and tongue [13]. The purpose of this study was to determine the colonization of *Candida* spp. in patients with PMMA and Cr–Co prosthetic appliances, as well as the salivary flow rate, and oral signs and symptoms.

## 2. Materials and Methods

### 2.1. Participants

The investigation included a total of 120 subjects from the Clinic of Dental Medicine, Clinical Hospital Center, Rijeka, Croatia. Subjects were divided into groups according to the type of material used for manufacturing of the prosthetic appliance. Group 1 (40 subjects) included the patients with polymethyl methacrylate (PMMA)-based denture in the upper jaw, with or without the similar appliance in the lower jaw. Group 2 (40 subjects) included the patients who wore removable denture with a cast Co–Cr metallic framework in the upper jaw, regardless of the type of appliance in the lower jaw. Finally, group 3 (40 subjects) comprised of the patients with no prosthetic appliances in their mouths, and served as the control group. All subjects had their remaining natural teeth restored and/or healthy. The groups were matched for age and gender. Demographic data for all subjects are listed in Table 1. This investigation excluded those patients who had been taking antimicrobial therapy within the previous 30 days, smokers, patients with systemic diseases, and immunologically compromised patients. Subjects were informed of the aims and purpose of the investigation and signed the informed consent for participation. This investigation was also previously approved by both the Ethics Committee of the Clinical Hospital Center Rijeka and Faculty of Dental Medicine in Rijeka, Croatia. 

### 2.2. Sample Collection

Detection of colonization and identification of *Candida* species in oral cavity.

The quantity of *Candida* colonizing the oral cavity was determined by using the concentrated oral rinse method. Before samples were collected, patients rinsed their mouths with sterile saline in order to remove food residue. Subjects then rinsed the mouth with 10 mL of sterile saline, and thus obtained samples were collected in sterile containers. Each sample was centrifugated at 2300× *g* for 20 min. The supernatant was removed and the resulting deposit was resuspended in 500 µL of sterile saline. Next, 100 µL of the concentrate was inoculated immediately onto Chromagar Candida (CHROMagar, Paris, France) in duplicate. Plates were incubated at 37 °C for 48 h according to the manufacturer guidelines. In cases of *Candida* overgrowth, the content was diluted 10 times, and 100 µL of diluted concentrate was immediately inoculated onto Chromagar Candida [13]. The number of colonies was expressed as colony-forming units per milliliter (CFU/mL). *Candida* cell counts of > 600 CFU per ml in concentrated oral rinse samples were considered a positive result. Oral yeast colonization was defined as the presence of yeasts in the oral cavity while oral candidiasis was defined as presence of *Candida* spp. in the oral cavity together with oral signs and symptoms [13,14,20]. The agar is able to differentiate between five species of *Candida* (*C. albicans*, *C. krusei*, *C. glabrata*, *C. tropicalis*, and *Candida* other species). *C. albicans* by growth was identified as light green coloured smooth colonies, *C. tropicalis* as blue to metallic blue coloured colonies, *C. glabrata* colonies appear as mauve-brown smooth colonies, while *C. krusei* appear as pink, fuzzy colonies. Isolates that produced white to mauve colonies were considered for *Candida* other species.

### 2.3. Determination of Salivary Flow Rate

Whole unstimulated saliva was measured by sialometry [21]. After the subjects swallowed the saliva, they started its collection by using a funnel and graduated tube (Copan, Zagreb, Croatia). During this procedure, subjects were comfortably seated with their head slightly tilted forward. Salivary samples were collected for 10 min. The measured values were expressed as milliliters per minute. At the time of saliva collection, subjects retained their prosthetic appliances in their mouth. Values of flow rate ≥ 0.36 mL per minute were regarded as normal, decreased flow rate ranged from 0.16 to 0.35 mL per minute, while hyposalivation was defined with flow rate < 0.15 mL per minute [22].

### 2.4. Clinical Examination

Clinical examination included an examination of oral mucosa and prosthetic appliance (denture). The inspection of the oral cavity was performed in the systematical procedure. Removable dentures were removed before examination of the oral mucosa. Clinical examination was used to describe the state of oral mucosa according to Toyama et al. [13]. Patients were examined for redness of oral mucosa, redness of the tongue, burning of tongue, taste disorder, tongue coating, and dry mouth. Redness of oral mucosa was graded in the following manner—negative: no redness on the oral mucosa; slight: localized redness areas without ulcerations; moderate: redness of the whole oral mucosa without ulcerations; severe: ulcerations with or without bleeding. Redness of the tongue was graded as—negative: less than 1/3 of the tongue showing slight redness; slight: about 2/3 of the tongue showing slight redness or about 1/3 of the tongue. Burning of tongue was graded using the visual analog scale—negative: 0 mm; slight: 1 mm; moderate: 30 mm; severe: over 54 mm. Taste disorder was graded as—negative: no change in taste; slight: altered taste but no change in diet; moderate: altered taste with change in diet, or noxious or unpleasant taste; severe: loss of taste. Tongue coating was graded as—negative: less than 1/3 of the tongue slightly coated; slight: about 2/3 of the tongue slightly coated or about 1/3 of the tongue thickly coated; moderate: about 2/3 of the tongue thickly coated; severe: more than 2/3 of the tongue thickly coated. Dry mouth was graded as—negative: non-dry; slight: saliva shows viscosity; moderate: saliva showing tiny bubbles on tongue; severe: dry tongue without viscosity, little or no saliva. 

Statistical analysis was performed with MedCalc Statistical Software version 19.1.7 (MedCalc Software Ltd., Ostend, Belgium; https://www.medcalc.org; 2020; accessed on 17 May 2021) and SPSS (IBM Corp. Released 2013. IBM SPSS Statistics for Windows, Version 21.0. Armonk, NY, USA: IBM Corp.). Categorical data were expressed with absolute and relative frequencies. Differences between categorical variables were tested with the χ^2^ test and the Fisher exact test, when deemed necessary. Normality of distribution for numerical variables was tested with the Shapiro–Wilk test. Numerical data were expressed as the median and limitations of interquartile range. Differences in numerical variables between three independent groups were tested with the Kruskal Wallis test. The level of significance was set to *p* = 0.05.

## 3. Results

### 3.1. Participant Demographics

Subjects were divided into three groups, with no significant differences in age or gender. Results are presented in Table 1.

### 3.2. Oral Candida Colonization

Values above 600 CFU/mL of *Candida* spp. were found in all subjects with PMMA prosthetic appliances (100%), in 35 subjects with Cr-Co framework dentures (87%), and in 26 subjects in the control group (65%). Results are presented in Table 2. Statistically significant differences were demonstrated between all tested groups (*p* < 0.0001). 

### 3.3. Identification of Candida Species

Distribution of different *Candida species* is listed in Table 3. In removable PMMA denture wearers, *C. albicans*, *C. krusei*, *C. glabrata*, and *C. tropicalis* were identified, whereas in the Cr–Co framework denture wearers and control subjects, *Candida other species* was identified in addition to the aforementioned species. In PMMA denture wearers, the predominant species was *C. albicans* (97.5%), followed by *C. krusei* (40%), *C. tropicalis* (17.5%), and *C. glabrata* (7.5%). In subjects with Cr–Co framework dentures, the predominantly isolated species was *C.albicans* (77.5%), followed by *C. krusei* (15%), *C. glabrata* (12.5%), *C. tropicalis* (7.5%), and *Candida other species* (2.5%). In the control group, the following species were isolated: *C. albicans* (62.5%), followed by *C. krusei* (7.5%), *C. glabrata* (7.5%), *C. tropicalis* (2.5%), and *Candida* other species (2.5%). A statistically significant difference was demonstrated in the prevalence of *C. albicans* (*p* < 0.001) and *C.krusei* (< 0.001) between PMMA denture wearers and control groups. 

### 3.4. Salivary Flow Rate 

**Figure 1:**
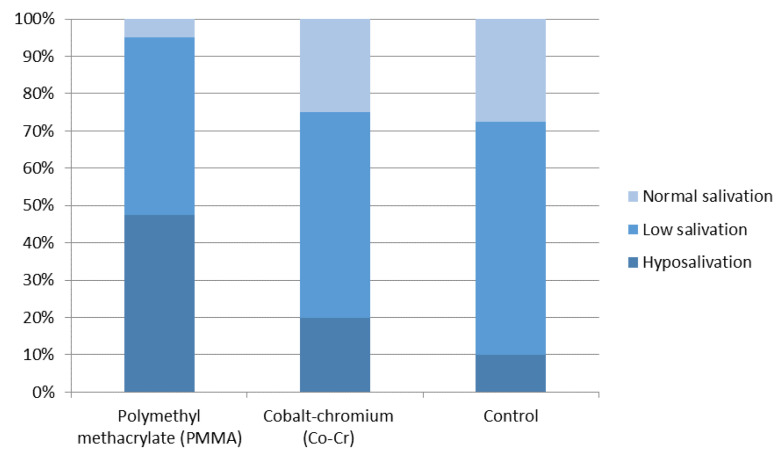
Salivary flow rate in investigated groups.

The distribution of salivary flow rate in subjects with PMMA removable denture wearers, Cr–Co framework denture wearers, and the control subjects is shown in Figure 1. Differences between the tested groups are statistically significant. The salivary flow rate is lowest in the PMMA group and is statistically different from both the Co–Cr and the control groups levels (*p* < 0.001).

### 3.5. Clinical Examination

Clinical examination included clinical oral signs and subjective symptoms. The presence and gradation of particular signs and symptoms are shown in Table 4. In subjects with PMMA denture wearers, a burning sensation of the tongue and oral muocsa dryness were significantly more frequent (both with *p* < 0.001) compared to the subjects with Cr–Co framework dentures, whereas red oral lesions were more frequently found in Cr–Co framework denture wearers (*p* < 0.001).

## 4. Discussion

The mere presence of prosthetic appliances changes the micro-environment of the oral cavity through the facilitation of *Candida* colonization and proliferation. Besides, the appliance itself adheres to the mucosal surface, thus preventing the physiological cleansing and normal saliva flow [9,23,24]. In this investigation, it was demonstrated that 93.8% of subjects with prosthetic appliances were positive for *Candida* spp. compared to 65% of the subjects who did not wear any appliance. Available data on *Candida* colonization in denture wearers show the percentage of positive findings in 80–100% of the subjects compared to 45–65% in denture-free subjects [25]. Many investigations confirmed that *Candida* is a common finding in a great number of patients with prosthetic appliances [6,26,27]. Prakash et al. [26] proved the presence of *Candida* in almost all subjects with prosthetic appliances, whereas only 52% of those who were denture-free had the same finding. *Candida* has the ability to adhere onto the surface and to form a complex biofilm which serves as an initial process for onset and progression of the disease, while the presence of a removable prosthetic appliance in the mouth accelerates colonization and biofilm formation. Initial adherence onto the denture surface also depends on physical properties of the material used for its fabrication. Porosity, free surface energy, hydrophobicity, and surface roughness all affect adherence; significant roles are also played by the method of denture fabrication, polymerization process, and surface modifications [28]. It has been demonstrated in in vitro conditions that the aforementioned material characteristics may determine the way biofilm is formed. Significantly greater growth of *Candida* was noted in PMMA-based materials compared to metal alloys [29]. In our investigation, significantly greater *Candida* colonization was found in subjects with PMMA-based prosthetic appliances. All subjects with PMMA removable prosthetic appliances were positive for *Candida* in their mouths. In patients with Cr–Co alloy frameworks, colonization was proven in 87% of subjects which was not significantly different from those subjects who did not wear any appliances. These results are comparable with those presented by Taha [30] who compared *Candida* colonization in patients with acrylic resin and metal framework dentures. Subjects with acrylic resin-based dentures more frequently showed *Candida* colonization compared to those with metal framework dentures, and they correlated these differences with characteristics of dental materials in question [26,31]. 

Oral cavity may harbor different species of *Candida*; however, *C. albicans* is certainly predominant [13,16,26,31]. It is believed that *C*. *albicans* makes up approximately 80% of all recovered *Candida* spp. and that those proportions remain unaltered in patients with and without clinical symptoms [16,26]. Identification of the exact species is of importance since different species have different abilities to cause infection, and certainly may cause variations in the patient’s response to therapy [13]. Beside *C. albicans*, commonly isolated species include *C*. *tropicalis*, *C. dubliensis*, and *C*. *glabrata*, whereas in some patients, it is not unusual to recover two or more different species at a time [31]. Our investigation showed that in PMMA-based denture wearers, the most commonly isolated species was *C. albicans*. This result is in accordance with other available investigations which also found that *C. albicans* was predominant [16,25,28]. Furthermore, *C. albicans* is predominantly isolated in metal framework denture wearers, although there was no significant difference compared to the control group. Beside *C. albicans*, the only other statistically significant difference was found for colonization with *C. krusei*. Nayak et al. [27] isolated—together with *C. albicans*—significantly higher levels of *C. glabrata*. Prevalence of non-albicans *Candida* spp., especially *C. glabrata* and *C. krusei*, has increased over recent years. Knowledge of the exact species is extremely important since these two species are known for their inadequate response to standard treatment protocols [32].

Colonization of the oral cavity by *Candida* is often correlated with a decreased salivation since it is known that this occurrence may change the quality of oral microflora [33,34]. This may be explained with a reduced rinsing of the oral cavity, alterations in acidity, and composition of saliva, as well as the diminished effect of anti-*Candida* factors that are usually secreted into the saliva [15]. Once *Candida* has colonized the oral mucosa surface due to the lack of its elimination with saliva and reduction in anti-*Candida* factors, it turns from non-pathogenic form of the yeast into a pathogenic hyphal form which facilitates penetration through the mucosal barrier [2,33]. In this investigation, we have shown differences in salivation between tested groups. Compared to the control group, salivary flow was significantly reduced in all denture wearers, although this was more pronounced in the group with PMMA-based dentures. This can be compared to the results of other available investigations [34]. 

*Candida* colonization, lack of saliva and its subsequent rinsing effect on the mucosa in close contact with the denture base, as well as the pressure emanating from the denture itself all create ideal conditions for the development of changes to oral mucosa and subjective symptoms. Patients who had more than 600 CFU/mL are exposed to a particular risk. Many investigations reported the development of red oral lesions related to *Candida* colonization [9,13,23,24]. Clinical examination performed within this investigation enabled detection of clinical oral signs and subjective symptoms. In patients with PMMA-based dentures, a burning sensation of the tongue and oral dryness were more frequent compared to metal framework denture wearers. However, pronounced red oral lesions were more frequently found in this latter group of patients (*p* < 0.001). This may be explained by better adherence of these metal-base dentures to oral mucosa [35]. Oral infections caused by *Candida* spp. are becoming more frequent. Primary causes include an increased number of patients with compromised immune system and systemic conditions, as well as favorable conditions in the oral cavity [2,32,36]. *Candida* spp. are normally harmless residents of the human mouth, but when conditions change, it may become pathogenic and cause diseases [37]. Various immunosuppressive conditions lead to changes in the oral cavity including oral candidiasis development, which is more prominent in immunocompromised patients than immunocompetent individuals [38]. Additionally, the presence of a prosthetic replacement can further aggravate the condition and encourage *Candida* reproduction [2,24]. Knowledge of these conditions, the type of *Candida*, the probability of colonization regarding a specific dental material, and clinical examination enable early recognition of changes, disturbances related to oral mucosa, and efficient prevention of oral candidiasis, which may consequently spread further into the organism. In this era when many people suffer from a compromised immunity, this is of particular importance.

## 5. Conclusions

Our investigation showed that subjects who have PMMA-based dentures more frequently exhibit *Candida* colonization, with *C. albicans* being the predominant species. Furthermore, those patients had a significantly decreased salivary flow rate which further facilitates the development of *Candida* colonies. Subjects with metal framework-based dentures were less prone to *Candida* colonization and had better values of salivary flow rate. Regardless of the material used for dentures, all patients need regular dental check-ups and good oral hygiene maintenance, both of which will provide a better quality of life for denture wearers and prevent the development of oral diseases. This investigation was limited to patients with different prosthetic appliances. Further investigation will be necessary to investigate oral *Candida* colonization in immunocompromised patients with prosthetic appliances. It is to be expected that, in this group of patients, the signs and symptoms identified in our study will be more pronounced. Our results can help prevent disease onset as much as possible in immunocompromised patients.

## Figures and Tables

**Table 1:** Demographic data of the subjects.

	Polymethyl Methacrylate (PMMA)	Cobalt–Chromium (Co–Cr)	Control	*p*
Subjects (*n*)	40	40	40	
Gender				
M	10	16	14	0.78
F	30	24	26	
Age				
Mean ± s.d ^1^	70.22 ± 9.46	65.2 ± 7.49	60.6 ± 9.61	0.09

^1^ standard deviation.

**Table 2:** Oral *Candida* colonization in participants.

		Number (%)		
	All Denture Wearers	Control	Total	*p* ^1^
*Candida* spp.				
Negative	5 (6.2)	14 (35)	19 (15.8)	<0.0001
Positive	75 (93.8)	26 (65)	101 (84.2)	
Total	80 (100)	40 (100)	120 (100)	

^1^ Fisher’s exact test.

**Table 3:** Distribution of *Candida species* in test groups.

	Polymethyl Methacrylate (PMMA)	Cobalt–Chromium (Co–Cr)	Control	Total	*p* ^1^	PMMA vs. Control *p* ^2^	Co–Cr vs. Control *p* ^2^
*C. albicans*							
No	1 (2.5)	9 (22.5)	15 (37.5)	25 (20.8)	0.001	<0.001	0.22
Yes	39 (97.5)	31 (77.5)	25 (62.5)	95 (79.2)			
*C. krusei*							
No	24 (60)	34 (85)	37 (92.5)	95 (79.2)	0.001	<0.001	0.48
Yes	16 (40)	6 (15)	3 (7.5)	25 (20.8)			
*C. glabrata*							
No	37 (92.5)	35 (87.5)	37 (92.5)	109 (90.8)	0.67	>0.99	0.71
Yes	3 (7.5)	5 (12.5)	3 (7.5)	11 (9.2)			
*C.tropicalis*							
No	33 (82.5)	37 (92.5)	39 (97.5)	109 (90.8)	0.06	0.06	0.62
Yes	7 (17,5)	3 (7,5)	1 (2,5)	11 (9,2)			
*Candida other* spp.							
No	40 (100)	39 (97.5)	39 (97.5)	118 (98.3)	>0.99	>0.99	>0.99
Yes	0	1 (2.5)	1 (2.5)	2 (1.7)			
*Total*	40 (100)	40 (100)	40 (100)	120 (100)			

^1^ χ^2^ test; ^2^ Fisher’s exact test.

**Table 4:** Oral signs and symptoms.

Oral Signs and Symptoms	Number (%) Subjects				*p* ^1^
	Polymethyl Methacrylate (PMMA)	Cobalt–Chromium (Co–Cr)	Control	Total	
Redness of oral mucosa					
negative	24 (60)	22 (55)	38 (95)	84 (70)	<0.001
slight	13 (32.5)	13 (32.5)	2 (5)	28 (23.3)	
moderate	3 (7.5)	4 (10)	0	7 (5.8)	
severe	0	1 (2.5)	0	1 (0.8)	
Redness of the tongue					
negative	37 (92.5)	38 (95)	40 (100)	115 (95.8)	0.37
slight	3 (7.5)	2 (5)	0	5 (4.2)	
Burning of tongue					
negative	25 (62.5)	34 (85)	39 (97.5)	98 (81.7)	<0.001
slight	4 (10)	5 (12.5)	0	9 (7.5)	
moderate	7 (17.5)	1 (2.5)	1 (2.5)	9 (7.5)	
severe	4 (10)	0	0	4 (3.3)	
Taste disorder					
negative	38 (95)	37 (92.5)	37 (92.5)	112 (93.3)	>0.99
slight	1 (2.5)	2 (5)	1 (2.5)	4 (3.3)	
moderate	1 (2.5)	1 (2.5)	2 (5)	4 (3.3)	
Coated tongue					
negative	26 (65)	28 (70)	35 (87.5)	89 (74.2)	0.08
slight	11 (27.5)	10 (25)	3 (7.5)	24 (20)	
moderate	3 (7.5)	2 (5)	1 (2.5)	6 (5)	
severe	0	0	1 (2.5)	1 (0.8)	
Dry mouth					
negative	7 (17.5)	18 (45)	32 (80)	57 (47.5)	<0.001
slight	20 (50)	15 (37.5)	6 (15)	41 (34.2)	
moderate	10 (25)	7 (17.5)	1 (2.5)	18 (15)	
severe	3 (7.5)	0 (0)	1 (2.5)	4 (3.3)	
Total	40 (100)	40 (100)	40 (100)	120 (100)	

^1^ Fisher’s exact test.

## Data Availability

The data presented in this study are available on request from the corresponding author. The data are not publicly available due to ethical issues.
